# Differences in Physiological Responses of Two Oat (*Avena nuda* L.) Lines to Sodic-Alkalinity in the Vegetative Stage

**DOI:** 10.3390/plants9091188

**Published:** 2020-09-11

**Authors:** Liyun Liu, Nateetorn Petchphankul, Akihiro Ueda, Hirofumi Saneoka

**Affiliations:** 1 Graduate School of Integrated Science for Life, Hiroshima University, 1-4-4 Kagamiyama, Higashi-Hiroshima 739-8528, Japan; akiueda@hiroshima-u.ac.jp (A.U.); saneoka@hiroshima-u.ac.jp (H.S.); 2 Graduate School of Biosphere Science, Hiroshima University, 1-4-4 Kagamiyama, Higashi-Hiroshima 739-8528, Japan; 3 Tropical Agriculture International Program, Faculty of Agriculture, Kasetsart University, Chatuchak, Bangkok 10900, Thailand; nateetornpetch@gmail.com

**Keywords:** alkalinity tolerance, antioxidant enzymes, bicarbonate, naked oat, phosphoenolpyruvate carboxylase

## Abstract

Sodic-alkalinity is a more seriously limiting factor in agricultural productivity than salinity. Oat (*Avena nuda*) is a salt-tolerant crop species and is therefore useful in studying the physiological responses of cereals to alkalinity. We evaluated the differential effects of sodic-alkalinity on two naked oat lines, Caoyou1 and Yanke1. Seedlings of the two lines were exposed to 50 mM alkaline salt mixture of NaHCO_3_ and Na_2_CO_3_ (18:1 molar ratio; pH 8.5) for 2 weeks in a soil environment. Sodic-alkalinity exposure led the assimilation of abundant Na^+^ at similar concentrations in the organs of both lines. However, Caoyou1 showed much stronger growth than Yanke1, exhibiting a higher dry weight, total leaf area, and shoot height under sodic-alkalinity. Further analysis showed that Caoyou1 was more sodic-alkalinity tolerance than Yanke1. This was firstly because of differences in the oxidative stress defense mechanisms in leaves of the two lines. Antioxidant enzyme activities were either slightly elevated (catalase (CAT), ascorbate peroxidase (APX), guaiacol peroxidase (GP), glutathione reductase (GR)) or unaltered (superoxide dismutase (SOD)) in Caoyou1 leaves, but some enzyme (SOD, GPOX, GR) activities were significantly reduced in Yanke1. *An*APX1 transcript levels significantly increased in Caoyou1 under sodic-alkalinity conditions compared with Yanke1, indicating its better antioxidant capacity. Secondly, the related parameters of Mg^2+^ concentration, phosphoenolpyruvate carboxylase (PEPC) activity, and *An*PEPC transcript levels in the leaves showed significantly higher values in Caoyou1 compared with Yanke1. This demonstrated the effective utilization by Caoyou1 of accumulated HCO_3_^−^ in the irreversible reaction from phosphoenolpyruvate to oxaloacetate to produce inorganic phosphorus, which was elevated in Caoyou1 leaves under alkalinity stress. Overall, the results demonstrated that the greater sodic-alkalinity tolerance of Caoyou1 is the result of: (1) maintained antioxidant enzyme activities; and (2) a higher capacity for the phosphoenolpyruvate to oxaloacetate reactions, as shown by the higher PEPC activity, Mg^2+^ concentration, and total phosphorus concentration in its leaves, despite the lower soil pH.

## 1. Introduction

Soil sodic-alkalinity is an increasing environmental problem, leading to losses in agricultural production [1,2]. Previous studies have reported that saline-alkalinity affects over 800 million hectares of land globally, and 10% of cultivated soils are subject to sodic-alkalinity [3,4]. Saline-alkalinity is increasing at a global annual rate of 0.25 to 0.5 Mha; the Food and Agriculture Organization of the United Nations (FAO) predicts that there will be a 50% reduction in land available for agriculture by the year 2050 due to these stresses [1,2,5].

Generally, the negative effects of salinity or sodic-alkalinity on plant growth divide into two major stress factors: osmotic and ionic stress [6]. Osmotic stress is an immediate phase that inhibits water uptake, whereas ionic stress develops after toxic Na^+^ ions have been assimilated into plant tissue at a level above the tolerance threshold [4]. The main effect of an Na^+^ cellular influx is membrane depolarization, which facilitates K^+^ efflux from the cytosol [4,6]. Maintaining a higher K^+^ accumulation in plant tissue is therefore crucial to plant salinity or sodic-alkalinity tolerance [4]. More seriously, under sodic-alkalinity conditions, abundant NaHCO_3_ or Na_2_CO_3_ results in a high soil pH (above 8.5) and high soil bicarbonate (HCO_3_^−^) level [7]. A high HCO_3_^−^ level surrounding the roots directly inhibits the absorption of mineral elements, such as Mg^2+^ and phosphorus, and disrupts ion homeostasis in plant tissue [8,9]. Photosynthesis is potentially inhibited by reductions in photosynthetic pigments caused by lower Mg^2+^ assimilation, as well as by reduced CO_2_ intake due to P limitation [10,11]. Therefore, sodic-alkalinity exerts similar reactions to salinity but with the added effect of high soil pH and high soil HCO_3_^−^, inhibits plant growth than salinity.

However, although abundant HCO_3_^−^ leads to plant growth inhibition and agricultural production losses, it has positive functions when present in small amounts. HCO_3_^−^ plays a basic role in the cell pH status of all plant organisms and contributes to essential metabolic pathways and the total assimilation of inorganic carbon in plants [12]. After passively entering plant roots, HCO_3_^−^ is transported over long distances via xylem vessels to the leaf tissue, where it serves as a substrate for different carboxylases, including acetyl coenzyme A carboxylase (acetyl-CoA; EC 6.4.1.2) and phosphoenolpyruvate carboxylase (PEPC; EC 4.1.1.31) [12]. PEPC is found in all plants, where it is associated with photosynthesis. The HCO_3_^−^ anion initially participates in an irreversible reaction that produces oxaloacetate and inorganic phosphorus from phosphoenolpyruvate, which is catalyzed by PEPC in the presence of Mg^2+^ [12,13]. 

A further damaging effect on the growth and development of plants under abiotic stress, including sodic-alkalinity, is the induction of oxidative stress [14], which commonly occurs due to the rapid accumulation of reactive oxygen species (ROS) [15,16]. Cell membranes are damaged by ROS through lipid peroxidation, causing electrolyte leakage from the cells [17]. ROS also affect various biological structures, leading to protein oxidation, enzyme inhibition, and damage to DNA and RNA [17]. ROS toxicity means that plant sodic-alkalinity tolerance depends on the detoxification of excess ROS [17], for which antioxidants, including both non-enzymatic and enzymatic mechanisms, are required for alleviating oxidative stress [15,16]. The non-enzymatic antioxidants include glutathione, ascorbate, carotenoids, phenolic compounds, and non-protein amino acids. The antioxidant enzymes include superoxide dismutase (SOD), catalase (CAT), ascorbate peroxidase (APX), glutathione reductase (GR), and guaiacol peroxidase (GP) [15,16,18], of which the first three are among the major ROS-scavenging antioxidant enzymes [17]. SOD can be found in almost all cellular compartments and catalyzes the dismutation of superoxide anions to hydrogen peroxide [16,17,19]. CAT and APX belong to two different classes of hydrogen peroxide-scavenging enzymes because of their different affinities for hydrogen peroxide [17]. APX has a high affinity for hydrogen peroxide and provides a crucial role in controlling its levels in different plant tissue compartments [15,17]. CAT is present in peroxisomes and is indispensable in hydrogen peroxide detoxification under abiotic stress [17]. 

Oat (*Avena sativa*) is classified into two main types, covered oat and naked oat (*Avena nuda* L.) [20]. Naked oat has been grown for centuries in China and the grain of naked oat lacks the fibrous outer husk [20]. Naked oat is a cereal crop that is rich in dietary fiber and kernel beta-glucan content, and is classified as being moderately salt-tolerant [14,21]. Compared with other crops, oat exhibits marked physiological traits when exposed to salinity, and is therefore useful as a study species in examining the physiological responses of cereal crops to sodic-alkalinity, which is more seriously limiting than salinity to agricultural productivity. However, oat genotypes such as Vao-9, Baiyan5, 16SA120850, 153ND121147, 121OA1432-5, and 5SA120093, also differ in their physiological responses to salinity or sodic-alkalinity [22,23]. Yanke1 and Caoyou1 are two lines of naked oats; Yanke1 is an edible oat line bred from 8115-1-2 and Jian1, and Caoyou1 is a grass oat line bred from 578 and Hebo1. The lower tolerance to sodic-alkalinity of the oat line Yanke1 compared with other lines has been found to depend only on photosynthetic parameters [24]. There is no currently available information on the sodic-alkalinity tolerance of Caoyou1, a line bred to exhibit high seed quality. 

In order to understand the adaptations to sodic-alkalinity of the two oat lines Caoyou1 and Yanke1, we examined how their growth, water status, K^+^ and Na^+^ assimilation, and antioxidative enzyme activity responded under sodic-alkalinity conditions. Additionally, we also examined the level of irreversible conversion from phosphoenolpyruvate to oxaloacetate in both lines by determining their Mg^2+^ and total phosphorus concentrations, and PEPC activity in the leaves.

## 2. Results

### 2.1. Plant Growth

**Figure 1:**
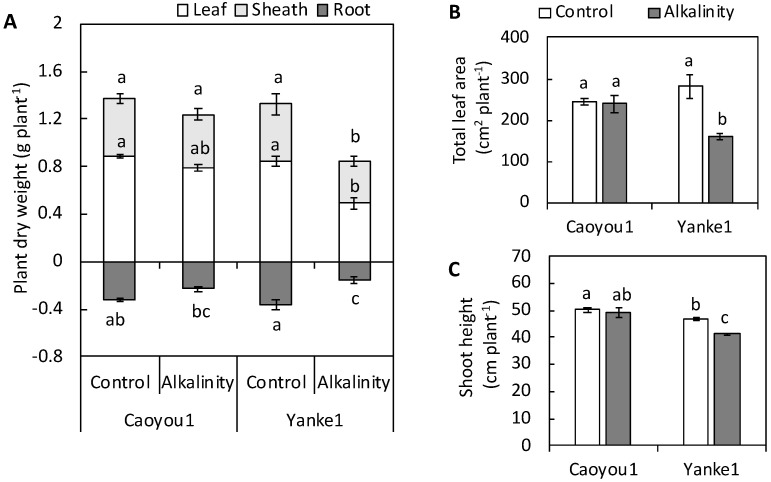
Effects of sodic-alkalinity on the (**A**) plant dry weight, (**B**) total leaf area, and (**C**) shoot height of the two oat lines Caoyou1 and Yanke1 after two weeks of treatment. Values are means of four replicates ± standard error. Different lower-case letters in the figures indicate significant differences between the treatments for both plant lines at *p* ≤ 0.05.

The results of subjecting the two oat lines Caoyou1 and Yanke1 to sodic-alkalinity conditions revealed that sodic-alkalinity clearly inhibited growth in Yanke1, but did so only in the root dry weight (DW) of Caoyou1 (Figure 1). Under sodic-alkalinity, the DW of the leaf, sheath, and root tissues of Yanke1 decreased significantly by 41.7, 28.3, and 55.8%, respectively, but DW reduction in Caoyou1 was only pronounced in the root tissues (27.5%) (Figure 1A). The total leaf area in Yanke1 was decrease by 43.2% under sodic-alkaline conditions when compared with the control, but this reduction was not showed in Caoyou1 (Figure 1B). Shoot height decreased by 12.1% in Yanke1 under sodic-alkaline conditions when compared with the control, but this reduction was not pronounced in Caoyou1 (Figure 1C).

### 2.2. Leaf Relative Water Content and Leaf Osmotic Potential

**Figure 2:**
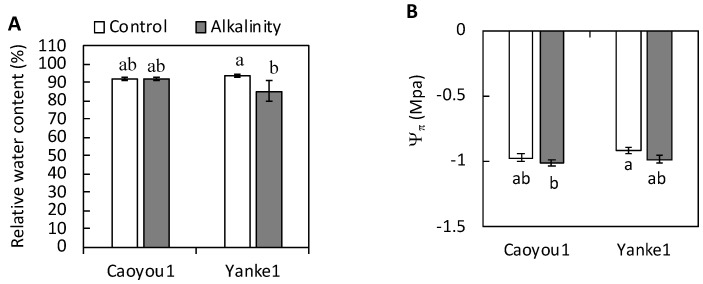
The relative water content (**A**) and osmotic potential (Ψ_π_) (**B**) in the leaves of the oat lines Caoyou1 and Yanke1 grown under control and sodic-alkalinity conditions for two weeks. Values are means of four replicates ± standard error. Different lower-case letters in the figures indicate significant differences between the treatments for both plant lines at *p* ≤ 0.05.

The results of measuring leaf relative water content (RWC) and osmotic potential (Ψ_π_) using leaf tissues in order to estimate water loss in the two lines (Figure 2) indicated that whereas Caoyou1 maintained a favorable tissue water status under sodic-alkalinity stress, a reduction in tissue water status was observed in Yanke1. Under sodic-alkalinity conditions, RWC in Yanke1 decreased by 9% when compared with the control, but there was no such reduction in Caoyou1 (Figure 2A). Ψ_π_ values were maintained similar levels in both lines under sodic-alkalinity stressed compared with the control plants (Figure 2B).

### 2.3. K^+^, Na^+^, and K^+^/Na^+^ Ratio in Leaf, Sheath, and Root Tissues

Sodic-alkalinity led to significant increases in the Na^+^ concentration in all the tissue samples examined from both lines, with no difference between them (Table 1). Sodic-alkalinity markedly decreased the K^+^ concentration in all tissue samples from both lines (Table 1), with no difference between them in the concentration in the leaf and sheath tissues, whether extracted from the control or sodic-alkalinity experiments. However, a significant higher root K^+^ concentration in root tissue was noted in Caoyou1 compared with Yanke1 under sodic-alkalinity conditions. The K^+^ concentration decreased by 30.3% in Yanke1 roots, but decreased by 18.9% in Caoyou1 roots (Table 1). Sodic-alkalinity led to a significant decrease in the K^+^/Na^+^ ratio in all the different tissues and was not different between the two lines (Table 1).

### 2.4. Mg^2+^ and Total P Concentrations in Leaf Tissue

**Figure 3:**
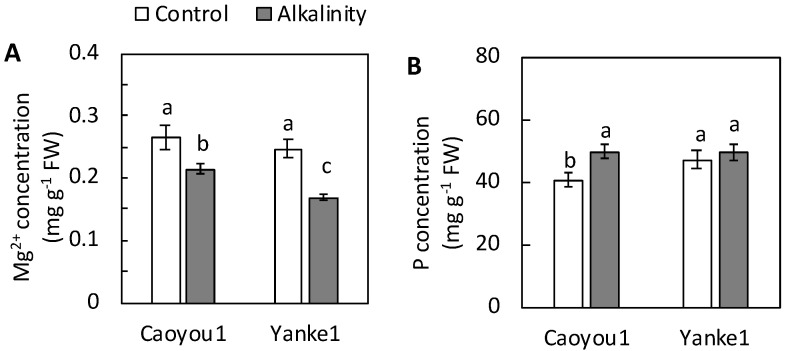
Effects of sodic-alkalinity on the Mg^2+^ concentration (**A**) and total P concentration (**B**) in the leaves of the two oat lines Caoyou1 and Yanke1 after two weeks of treatment. Values are means of four replicates ± standard error. Different lower-case letters in the figures indicate significant differences between the treatments for both plant lines at *p* ≤ 0.05.

The Mg^2+^ concentration in the leaf tissue of both lines decreased significantly under sodic-alkalinity conditions compared with the control but was less apparent in Caoyou1 (19.2%) than in Yanke1 (31.5%) (Figure 3A). Sodic-alkalinity did not alter the total P concentration in the leaves of Yanke1, but increased the total P concentration by 22.0% in Caoyou1 leaves, compared with the control (Figure 3B).

### 2.5. Phosphoenolpyruvate Carboxylase Activity and Phosphoenolpyruvate Carboxylase-Coding Gene Expression in Leaf Tissue

**Figure 4:**
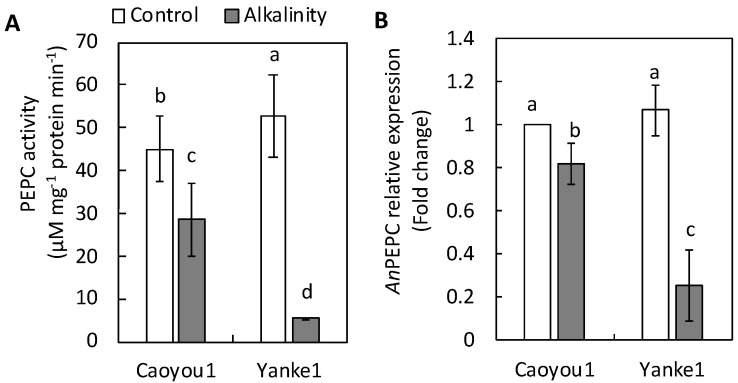
Phosphoenolpyruvate carboxylase (PEPC) activity (**A**) and relative expression of the phosphoenolpyruvate carboxylase encoding gene (*An*PEPC) (**B**) in the leaves of the oat lines Caoyou1 and Yanke1 grown under control and sodic-alkalinity conditions for two weeks. Values are means of four replicates ± standard error. Different lower-case letters in the figures indicate significant differences between the treatments for both plant lines at *p* ≤ 0.05.

PEPC activity in the leaf tissue of both lines decreased under sodic-alkalinity conditions compared with the control, but this reduction was less apparent in Caoyou1 (36.4%) than in Yanke1 (88.9%) (Figure 4A). Sodic-alkalinity induced a repression in expression of the PEPC-coding gene, *An*PEPC, by over 80% and 20% in Caoyou1 and Yanke1, respectively (Figure 4B).

### 2.6. Antioxidant Enzyme Activity in Leaf Tissue

**Figure 5:**
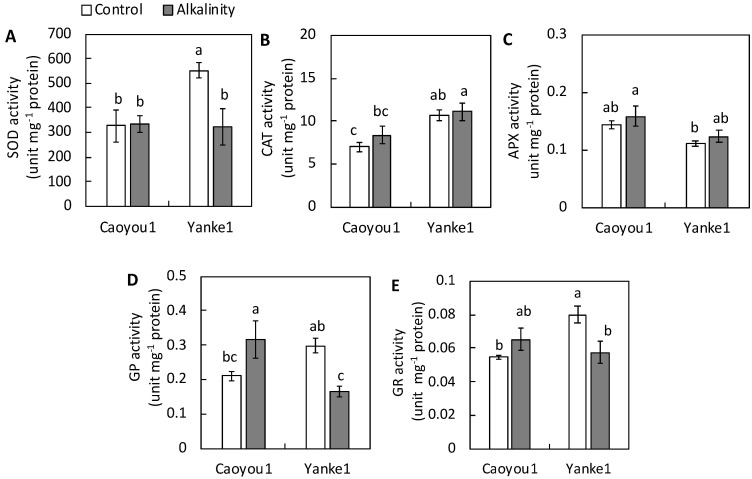
Activities of the antioxidant enzymes (**A**) superoxide dismutase (SOD), (**B**) catalase (CAT), (**C**) ascorbate peroxidase (APX), (**D**) guaiacol peroxidase (GPOX), and (**E**) glutathione reductase (GR) in the leaves of the oat lines Caoyou1 and Yanke1 grown under control and sodic-alkalinity conditions for two weeks. Values are means of four replicates ± standard error. Different lower-case letters in the figures indicate significant differences between the treatments for both plant lines at *p* ≤ 0.05.

The assays used to measure SOD, CAT, APX, GP, and GR enzyme activity in the leaf tissue of both lines enabled their antioxidant capacity to be estimated (Figure 5). SOD is an important antioxidant enzyme that catalyzes the dismutation of O_2_^−^ to H_2_O_2_. SOD enzyme activity in Yanke1 leaf tissue decreased by 41.5% under sodic-alkalinity conditions compared with the control, but no reduction was observed in Caoyou1 (Figure 5A). CAT and APX enzymes play vital roles in plant defense against the oxidative stress caused by hydrogen peroxide. CAT and APX enzyme activities were maintained at similar levels in both lines under sodic-alkalinity conditions compared with their controls (Figure 5B,C). GP enzyme activity decreased by 44.2% in Yanke1 under sodic-alkalinity conditions, but increased by 50.5% in Caoyou1 compared with control plants (Figure 5D). Similarly, GR enzyme activity decreased by 19.5% in Yanke1 under sodic-alkalinity conditions but remained at a similar level in Caoyou1, compared with control plants (Figure 5E).

### 2.7. Expression Analysis of Antioxidant Enzyme-Coding Genes

**Figure 6:**
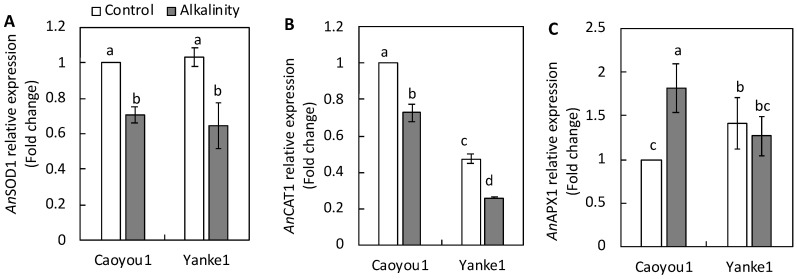
Relative expression of the genes (**A**) copper-zinc superoxide dismutase (*An*SOD1), (**B**) catalase 1 (*An*CAT1), and (**C**) ascorbate peroxidase (*An*APX1) in the leaves of the oat lines Caoyou1 and Yanke2 grown under control and sodic-alkalinity conditions for two weeks. Values are means of four replicates ± standard error. Different lower-case letters in the figures indicate significant differences between the treatments for both plant lines at *p* ≤ 0.05.

The relative expression of genes involved in plant adaptation to sodic-alkalinity through their antioxidant capacity is shown in Figure 6. Under sodic-alkalinity conditions, the expression of the SOD enzyme-coding gene, *An*SOD1, was repressed in both lines (Figure 6A). Sodic-alkalinity induced a repression in the expression of the CAT enzyme-coding gene, *An*CAT1, by over 70% and 50% in Caoyou1 and Yanke1, respectively (Figure 6B). Sodic-alkalinity induced expression of the APX enzyme-coding gene, *An*APX1, in Caoyou1 by over 1.8-fold, compared with a maintained expression in Yanke1 leaf tissue (Figure 6C).

## 3. Discussion

The current study was conducted to determine differences in how sodic-alkalinity affected two oat lines, Caoyou1 and Yanke1. Caoyou1 was found to be more clearly tolerant of sodic-alkalinity than Yanke1; Caoyou1 maintained a higher plant biomass, total leaf area, shoot height (Figure 1), leaf water content (Figure 2), Mg^2+^ concentration (Figure 3A), PEPC activity (Figure 4A), and antioxidative capacity (Figure 5), in addition to an increased total phosphorus concentration(Figure 3B) in the leaves. The sodic-alkalinity stress tolerance of numerous plants is associated with a low Na^+^ concentration in plant tissue [4]. However, in the current study, both lines were found to show the same Na^+^ concentration in their leaves, sheathes, and roots (Table 1), with Caoyou1 showing greater tolerance than Yanke1. There is therefore an urgent requirement to explain the mechanisms by which Caoyou1 tolerates sodic-alkalinity, but which appear to be absent in Yanke1. The results of the current study indicate some credible explanations: firstly, Caoyou1 maintained a higher water uptake ability in its leaves and higher K^+^ accumulation in its roots than Yanke1 under sodic-alkalinity conditions; secondly, Caoyou1 showed a greater capacity to maintain parameters supporting photosynthesis than Yanke1; and thirdly, the leaves of Caoyou1 showed a greater ability to maintain their antioxidative ability than in Yanke1.

### 3.1. Water Uptake Capacity in Leaves and K^+^ Accumulation in Roots

Sodic-alkalinity normally leads to tissue water deficiency in plants, and RWC and Ψ_π_ are frequently used to evaluate this [25,26,27], as reductions in these two parameters under sodic-alkalinity conditions indicate water deficiency. Low values of RWC and Ψ_π_ usually correspond to cell dehydration, decreased cell expansion, and decreased leaf area. In the current study, Caoyou1 maintained RWC levels and showed a lower reduction in leaf Ψ_π_ compared with Yanke1 under sodic-alkalinity conditions (Figure 2), indicating that the former suffered lower water deficiency. Consequently, stability in the water uptake of Caoyou1 enabled its total leaf area to be maintained (Figure 1B), in contrast with the reduction in total leaf area in Yanke1. The conserved total leaf area in Caoyou1 provided a significant surface area for photosynthate production when subject to sodic-alkalinity, compared with Yanke1. Therefore, the ability of Caoyou1 to sustain its leaf water status under sodic-alkalinity conditions may be one source of evidence explaining its greater sodic-alkalinity tolerance compared with Yanke1. 

Na^+^ is the main toxic ion impairing plant growth under alkalinity conditions [4]. The most significant plant adaptation to cope with Na^+^ toxicity is the ability to promote K^+^ accumulation in plant tissue [4]. Therefore, lines exhibiting a higher root K^+^ concentration, such as Caoyou1, could be better adapted to manage under sodic-alkalinity.

### 3.2. Total P and Phosphoenolpyruvate Carboxylase Activity in Leaf Tissue

Under sodic-alkalinity conditions, the high pH environment surrounding the roots directly decreases phosphorus availability, causing it to become deficient in plants; this is a major factor in biomass reduction in plants subject to sodic-alkalinity [9,28,29]. However, in the current study, Caoyou1 showed an increased total phosphorus concentration in its leaves, suggesting that phosphorus was transported effectively from the soil to its roots and then to its leaves (Figure 3B). This increased phosphorus concentration may act to increase the CO_2_ intake available for photosynthesis in the leaves of Caoyou1 under sodic-alkalinity conditions when compared with the unchanged total P in Yanke1, thereby enhancing its sodic-alkalinity tolerance. Moreover, the higher Mg^2+^ assimilation may enhance the availability of both photosynthetic pigments and phosphorus for phosphate metabolism (Figure 3A) and help prevent inhibition of photosynthesis, thereby effectively alleviating any harmful effects on plant growth in Caoyou1 (Figure 1A). 

PEPC is a tightly controlled cytosolic enzyme located at the core of plant carbon metabolism [30]. With Mg^2+^, this enzyme irreversibly catalyzes the conversion of phosphoenolpyruvate and HCO_3_^−^ to inorganic phosphate and oxaloacetate, the latter of which is further converted into organic acids [13,30]. These organic acids are then released into the rhizosphere and alter its pH, thereby acting in parallel with HCO_3_^−^-induced plant growth inhibition [12]. In the current study, reduction in PEPC activity in the leaves was significantly lower in Caoyou1 than in Yanke1 under sodic-alkalinity conditions (Figure 4A), indicating that Caoyou1 may better retain the reactions required to form inorganic phosphate and oxaloacetate. Consequently, the promotion of a lower pH by Caoyou1 (Table 2) in the soil surrounding Caoyou1 may be due to the release of organic acids from roots under sodic-alkalinity conditions, compared with the unchanged soil pH of Yanke1. This might improve phosphorus availability in the rhizosphere, thereafter increasing the total leaf phosphorus concentration in Caoyou1 (Figure 3B). 

Zhang et al. [31] reported that the introduction of the maize PEPC gene into wheat improved wheat photosynthetic characteristics when compared with untransformed plants, and was a viable means of improving wheat biomass. The authors also found that expression of the PEPC gene was markedly higher in the transgenic line than in untransformed lines. In the current study, the higher *An*PEPC transcription level in the leaves of Caoyou1 under sodic-alkalinity conditions suggests that *An*PEPC may play an important role in mechanisms defending against reductions in photosynthesis.

### 3.3. Differential Antioxidative Capacity in Leaf Tissue

Under abiotic stress, overreduction of photosynthetic electron transport activity often causes an unfavorable dissipation of excess light energy. This in turn leads to overproduction of ROS [15,25], of which the superoxide anion is the most pervasive of those generated in the electron transport chains associated with photosynthesis [15,16,32]. This radical causes membrane damage in plant tissues, and must be rapidly removed from the photosynthetic system to avert membrane peroxidation. SOD activity normally regulates the removal of superoxide anions by dismutating them to hydrogen peroxide and oxygen [32]. The current study did not evaluate superoxide anions because of the certainty of ROS overproduction in plants under abiotic stress. However, SOD activity levels associated with superoxide anion detoxification in the leaves of Caoyou1 remained unchanged under sodic-alkalinity conditions compared with the control, but significantly decreased in Yanke1 leaves (Figure 5A).

Under natural conditions, overproduced hydrogen peroxide also leads to oxidative damage and must be removed immediately; to achieve this, plants often employ other antioxidant enzymes (CAT, APX, GP, and GR) to avert membrane peroxidation. CAT is indispensable to this detoxification process; it functions in concert with GP to scavenge hydrogen peroxide, thereby maintaining intracellular hydrogen peroxide at low levels [17]. Under sodic-alkalinity conditions in the current study, the maintained CAT activity and significant increase in GP activity increased the turnover rate of hydrogen peroxide scavenging in the leaves of Caoyou1 but not Yanke1 (Figure 5B,D). However, CAT shows a relatively low affinity for hydrogen peroxide, and so is insufficient on its own in regulating it to non-toxic levels [15,16]. In this case, the high affinity of APX for hydrogen peroxide provides a crucial role in controlling its levels in different plant tissue compartments [15,17]. APX functions in concert with GR to convert hydrogen peroxide to water in the ascorbate-glutathione cycle [17]. In the current study, the increase in APX enzyme activity was similar between the two lines (Figure 5C). However, the slightly increased GR enzyme activity (Figure 5E) expedited ascorbate-glutathione cycle reactions in the leaves of Caoyou1 to a greater level than Yanke1 under sodic-alkalinity conditions, thereby maintaining a low level of cellular hydrogen peroxide. These results suggested SOD, CAT, APX, GP, or GR maintained the ROS scavenging capacity in Caoyou1 leaves under sodic-alkalinity conditions but that SOD, GP, and GR activity was lacking in Yanke1 leaves. Therefore, differences in antioxidant enzyme activity in the leaves may be important to sodic-alkalinity tolerance in Caoyou1. 

Kong et al. [33] reported that the oat copper-zinc superoxide dismutase 1 (*An*SOD1), catalase 1 (*An*CAT1), and ascorbate peroxidase 1 (*An*APX1) genes showed altered transcript levels and a strongly suppressed expression in oat seeds subject to long-term storage. Similarly, the transcript levels of these same three genes were suppressed in the leaves of Yanke1 under sodic-alkalinity conditions (Figure 6), suggesting that they did not contribute to the total SOD, CAT, and APX enzyme activities. However, Wu et al. [34] reported that a salt-tolerant oat line expressed higher transcript levels of antioxidant enzyme genes than a salt-sensitive line in response to salinity. In the current study, *An*APX1 transcript levels increased 1.5-fold in the leaves of Caoyou1 under sodic-alkalinity conditions (Figure 6C), suggesting that *An*APX1 may play a role in the defense mechanism against sodic-alkalinity-induced oxidative stress in this line.

## 4. Materials and Methods 

### 4.1. Plant Materials, Growth Conditions, Alkalinity Treatment, and Post-Treatment Soil pH 

Naked oat (*Avena nuda*; Caoyou1 and Yanke1) seeds were collected from the Inner Mongolia Agricultural University, China, and then sown in a seed bed in the greenhouse of the School of Applied Biological Science, Hiroshima University, Japan. Nine days after germination, sixteen uniform seedlings were selected for each line and then transferred to eight 2-L pots filled with soil containing substrate, granite, and perlite (2:2:1, *v*/*v*/*v*). Each pot contains two seedlings. The seedlings per pots were irrigated regularly per pot with a nutrient solution of 1 mM KH_2_PO_4_, 0.2 mM MgSO_4_, 1.3 mM KNO_3_, 0.3 mM Ca(NO_3_)_2_, 0.6 mM K_2_SO_4_, 3 mM NH_4_NO_3_, 10 µM Fe-EDTA, 4.6 µM MnCl_2_, 0.4 µM CuSO_4_, 57.8 µM H_3_BO_3_, 0.15 µM MoO_3_, and 1.0 µM ZnSO_4_. Twenty-day-old seedlings were subjected to either 0 (control) or 50 mM sodic-alkalinity (NaHCO_3_:Na_2_CO_3_ = 18:1 molar ratio; pH 8.5) for two weeks.

After treatment, the crude soil was oven-dried at 70 °C for 3 days and then sieved thoroughly. Then, approximately 20 g soil was mixed with 50 mL deionized water for 2 days. After filtration, the soil pH of the filtrate was determined using a pH meter (AS700 pH meter, AS ONE SHANGHAI Corp., Shanghai, China). Details of post-treatment soil pH are presented in Table 2.

### 4.2. Measurement of Shoot Height, Total Leaf Area, and Dry Weight 

Shoot height was measured based on the shoot length from the soil surface to shoot top for the two lines, under both control and sodic-alkalinity conditions, before harvesting. Subsequently, a leaf area meter (AMM-5, Hayashi-Denko Co., Ltd., Tokyo, Japan) was used to measure total leaf area for both lines [32]. The roots of the same plants with soil were isolated from the pots and washed carefully to separate roots [35]. Then, the leaves, sheathes, and roots were oven-dried at 70 °C for three days to record the dry weight, while young leaf tissue samples were flash-frozen in liquid nitrogen and stored at −80 °C until required for analysis.

### 4.3. Measurement of Relative Water Content and Osmotic Potential in Leaf Tissue

The leaf relative water content (RWC) of the first fully expanded (counted from the top) leaf per plant was measured according to the method of Mekawy et al. [36]. Briefly, the leaves were cut into 3 cm long segments and the fresh weight (FW) was recorded. The leaf samples were then soaked in fresh distilled water for 24 h under light, and thereafter were gently wiped to remove excess water, and weighed again to determine the fully turgid weight (TW). The samples were next oven-dried at 70 °C for three days, and the DW was recorded. The RWC was then determined using the following formula: RWC = 100 × (FW − DW)/(TW − DW). The second fully expanded leaf was then selected to measure osmotic potential (Ψ_π_). Each leaf sample was immediately stored in a 1-mL microtube with a small hole at the bottom. The 1 mL microtube was then placed in a 5-mL microtube and centrifuged at 3000 rpm for 10 min to collect cell sap from the leaf sample. The leaf Ψ_π_ value was recorded by measuring cell sap pressure using a pressure osmometer (Wescor 5500, Wescor Inc., Logan, UT, USA).

### 4.4. Determination of Na^+^ and K^+^ in Different Plant Organs

Approximately 50 mg of fresh plant tissue (leaf, sheath, and root) were digested with nitric acid (HNO_3_) and hydrogen peroxide (H_2_O_2_) (*v*/*v*, 2:1). After digestion, the Na^+^ and K^+^ concentrations were recorded using a flame photometer (ANA-135, Eiko Instruments Inc., Tokyo, Japan).

### 4.5. Determination of Mg^2+^ and Total P in Leaf Tissue

Approximately 50 mg fresh leaf sample was digested with HNO_3_ and H_2_O_2_ (*v*/*v*, 2:1). After digestion, Mg^2+^ was measured using an atomic absorption spectrometer (AA-6200, Shimadzu Corporation, Kyoto, Japan), and the total P concentration was determined using a UV-spectrophotometer (U-3310, Hitachi Co., Ltd., Tokyo, Japan) following the molybdenum reaction solution method suggested by Chen et al. [37].

### 4.6. Measurement of Phosphoenolpyruvate Carboxylase Activity in Leaf Tissue

Approximately 0.5 g of frozen leaves were ground in liquid nitrogen and extracted using 5 mL of ice-cold extraction buffer (200 mM HEPES-NaOH (pH 7.0), 2% polyvinylpyrrolidone, 5 mM dithiothreitol, 10 mM MgCl_2_). Then, the homogenate was centrifuged at 10,000× *g* for 10 min; the supernatant was immediately used for determining the PEPC activity and the soluble protein concentration [38]. The 1-mL PEPC reaction mixture contained 50 mM HEPES-KOH buffer (pH 8.0), 2 mM phosphoenol pyruvate (PEP), 5 mM MgCl_2_, 2 mM KHCO_3_, 15% glycerol, 0.15 mM reduced nicotinamide adenine dinucleotide (NADH), 2 mM dithiothreitol, 5 units mL-1 malate dehydrogenase, and 10% PEPC extract. NADH oxidation was monitored at 340 nm, and PEPC activity was calculated using the NADH molar extinction coefficient (6.22 mM^−1^ cm^−1^). The soluble protein concentration in the PEPC extract was determined using a DC (detergent compatible) protein assay kit (Bio-Rad, Hercules, CA, USA). All processes were conducted following the manufacturer’s instructions.

### 4.7. Measurement of Antioxidant Enzyme Activity in Leaf Tissue

In order to analyze antioxidant enzyme activity, 0.5 g of frozen leaf tissue samples were ground in liquid nitrogen and extracted using a 5-mL ice-cold extraction buffer (25 mM potassium phosphate, 0.5 mM ethylenediaminetetraacetic acid (EDTA), 1 mM ascorbic acid, and 2% polyvinylpolypyrrolidone; pH 7.8). The homogenate was then centrifuged, and the supernatant was collected for determining the antioxidant enzyme activity and the soluble protein concentration [25]. 

The assay mixture (1 mL) used to determine SOD enzyme activity contained 50 mM potassium phosphate buffer (pH 7.8), 13 mM methionine, 0.1 mM EDTA, 2 mM riboflavin, 75 mM nitroblue tetrazolium, and 10% enzyme extract. After preparation, one set of the assay mixture was illuminated with 25-W fluorescent lamps for 15 min, and the other set was kept in the dark as a control. The post-reaction absorbance was measured at 560 nm; one unit of SOD activity was defined as the enzyme amount required to cause 50% inhibition of nitro blue tetrazolium photoreduction [39]. The 1 mL assay mixture used to determine CAT activity contained 50 mM potassium phosphate buffer (pH 7.0), 10 mM H_2_O_2_, and 5% enzyme extract. Decreases in H_2_O_2_ were monitored at 240 nm and CAT activity was expressed as mmol H_2_O_2_ consumed per minute [40]. One unit of CAT activity was defined as the amount of enzyme that decomposes 1 mmol H_2_O_2_ in 1 min of reaction time at 25 °C. The 1 mL assay mixture used to determine APX activity contained 25 mM phosphate buffer (pH 7.0), 0.1 mM H_2_O_2_, 0.1 mM EDTA, 0.25 mM ascorbic acid, and 10% enzyme extract. APX activity was calculated using the molar extinction coefficient for ascorbic acid (2.8 mM^−1^ cm^−1^) [41]. One unit of APX activity was defined as the amount of enzyme that decomposes 1 mmol ascorbic acid per minute at 25 °C. The 1 mL reaction mixture used to determine GR activity contained 40 mM potassium phosphate buffer (pH 7.5), 0.4 mM EDTA, 0.02 mM NADP, 0.78 mM glutathione, and 10% enzyme extract. NADPH (reduced form of NADP) oxidation was monitored at 340 nm, and GR activity was calculated using the molar extinction coefficient for NADPH (6.22 mM^−1^ cm^−1^) [42]. One unit of GR activity was defined as the amount of enzyme that decomposes 1 mmol NADPH per minute at 25 °C. The 1 mL reaction mixture for GP contained 70 mM potassium phosphate buffer (pH 7.0), 10 mM H_2_O_2_, 15 mM guaiacol, and 2% enzyme extract. GP activity was calculated using the molar extinction coefficient for tetraguaiacol (26.6 mM^−1^ cm^−1^) [43]. One unit of GP activity was defined as the amount of enzyme that produces 1 mmol tetraguaiacol per minute at 25 °C. The soluble protein concentration in the enzyme extract was determined using the DC protein assay kit (BioRad); all processes were conducted following the manufacturer’s instructions.

### 4.8. RNA Isolation and Real-Time Polymerase Chain Reaction

Total RNA was extracted from the leaf tissue of the control and stressed plants using a total RNA Extraction Kit (Plant) (RBC Bioscience, SciTrove, Tokyo, Japan), and first-strand cDNA was synthesized from 1 μg of extracted total RNA using a ReverTra Ace qPCR RT kit (Toyobo, Osaka, Japan). Then, a quantitative polymerase chain reaction (qPCR) was conducted using a Thunderbird SYBR qPCR Mix (Toyobo, Osaka, Japan) and StepOnePlus system (Applied Biosystems, Foster City, CA, USA) [44]. After initial denaturation at 95 °C for 1 min, the reaction was followed by 35 cycles of denaturation at 95 °C for 15 s with an extension at 58 °C for 60 s. The relative expression levels of different genes were calculated with the comparative 2^−∆∆CT^ method [45], using the actin gene as an internal control. The primer sequences used are listed in Table 3. 

### 4.9. Data Analysis and Statistics 

All data collected were subjected to one-way analysis of variance (ANOVA) using the IBM SPSS statistical package version 25 (IBM Corp., Armonk, NY, USA). Significance testing was performed using Duncan’s multiple range test at *p* ≤ 0.05. The values were means (± standard error; SE) of four replicates. 

## 5. Conclusions

In conclusion, the current study clearly exhibited that the two oat lines differed in sodic-alkalinity tolerance, with Caoyou1 being more tolerant than Yanke1. This difference was partially attributed to differences in antioxidant enzyme activity, which supported a more robust defense mechanism against oxidative stress in the leaves of Caoyou1 compared with Yanke1 under alkalinity conditions. The maintained ROS-scavenging potential in Caoyou1 compared with Yanke1 leaves could contribute to better photosynthetic characteristics in the former, due to the increased P concentration and higher Mg^2+^ assimilation and PEPC activity. Moreover, this difference in sodic-alkalinity tolerance in Caoyou1 is in part due to differences in leaf water status and root K^+^ accumulation, which were more pronounced in Caoyou1 compared with Yanke1. Overall, therefore, the results showed that Caoyou1 had a greater sodic-alkalinity tolerance than Yanke1; this greater ability was attributable to its superior water status, sustained antioxidative capacity, and higher rate of leaf PEPC activity compared with Caoyou1.

## Figures and Tables

**Table 1:** Na^+^ concentration, K^+^ concentration, and K^+^/Na^+^ ratio in the fresh leaves, sheathes, and roots of the oat lines Caoyou1 and Yanke1 under control and sodic-alkalinity conditions after treatment for two weeks. Values are means of four replicates ± standard error. Different lower-case letters in the table indicate significant differences between the treatments for both plant lines at *p* ≤ 0.05.

Measurements	Tissues	Caoyou1		Yanke1	
		Control	Sodic-Alkalinity	Control	Sodic-Alkalinity
Na^+^ (mg g^−1^ FW)	Leaf	0.02 ± 0.00 ^b^	2.95 ± 0.07 ^a^	0.01 ± 0.00 ^b^	2.93 ± 0.10 ^a^
	Sheath	0.02 ± 0.00 ^b^	2.59 ± 0.12 ^a^	0.02 ± 0.00 ^b^	2.44 ± 0.07 ^a^
	Root	0.10 ± 0.00 ^b^	2.42 ± 0.10 ^a^	0.10 ± 0.01 ^b^	2.29 ± 0.11 ^a^
K^+^ (mg g^−1^ FW)	Leaf	6.72 ± 0.17 ^a^	3.20 ± 0.07 ^b^	7.19 ± 0.54 ^a^	3.27 ± 0.19 ^b^
	Sheath	6.41 ± 0.09 ^a^	3.24 ± 0.11 ^b^	6.30 ± 0.04 ^a^	3.17 ± 0.16 ^b^
	Root	3.06 ± 0.14 ^a^	2.48 ± 0.08 ^b^	2.67 ± 0.09 ^ab^	1.86 ± 0.22 ^c^
K^+^/Na^+^	Leaf	416.69 ± 39.9 ^b^	1.08 ± 0.01 ^c^	574.45 ± 51.02 ^a^	1.12 ± 0.07 ^c^
	Sheath	314.60 ±1 0.23 ^b^	1.26 ± 0.06 ^c^	396.84 ± 10.22 ^a^	1.31 ± 0.10 ^c^
	Root	30.65 ± 1.60 ^a^	1.03 ± 0.06 ^b^	29.74 ± 2.26 ^a^	0.80 ± 0.07 ^b^

**Table 2:** Soil pH of the oat lines Caoyou1 and Yanke1 under control and sodic-alkalinity conditions after treatment for two weeks. Values are means of four replicates ± standard error. Different lower-case letters in the table indicate significant differences between the treatments for both plant species at *p* ≤ 0.05.

Lines	Treatments	Soil pH
Coyou1	Control	6.42 ± 0.02 ^d^
	Sodic-alkalinity	8.31 ± 0.02 ^b^
Yanke1	Control	6.53 ± 0.02 ^c^
	Sodic-alkalinity	8.48 ± 0.01 ^a^

**Table 3:** Primers used for quantitative real-time polymerase chain reaction RT-PCR.

Genes	Forward Primers (5′–3′)	Reverse Primers (5′–3′)
*An*SOD1	CACAAGCACTTCACAGGAACAGT	TGCCACTCTGAACATTTCATCAC
*An*CAT1	CAGGCTGGCGAGAGATTCC	AGCATCCGTGAGTGCATCAA
*An*APX1	GCTCCGTGAAGTAAGTGTTATCAAAC	CCTGGGAAGGTGCCACAA
*An*PEPC	AAGCTGCTGGGTCTCTTCAT	AGATGTACAACGAGTGGCCA
*An*actin	CCAGAGTCTAAGACGCAACC	CGTGAAAGAATGACCCAAAT
